# Start Early and See Inflammatory; Late, Nothing Save RAVE: How to Appreciate Radiation Proctitis as a Continuum

**DOI:** 10.1016/j.gastha.2022.11.001

**Published:** 2022-11-09

**Authors:** Martin Tobi, Irwin Bradley, Sumana Moole, Harvinder Talwar, Benita McVicker, Esperanza Kintanar, Paula Sochacki, Edgar Ben-Josef

**Affiliations:** 1 Department of R&D, Detroit VAMC, Detroit Michigan; 2 St. Lukes Hospital, Duluth, Minnesota; 3 Merus Gastroenterology, and Gut Health LLC, Suwansee Georgia; 4 Research Service, VA Nebraska-Western Iowa Health Care System, the University of Nebraska Medical Center, Omaha, Nebraska; 5 Department of Pathology, Ascension Michigan Providence Hospital, Rochester Michigan; 6 Department of Pathology, Detroit VAMC, Detroit Michigan; 7 Department of Radiation Oncology, Hospital of the University of Pennsylvania, Perelman Center for Advanced Medicine, Philadelphia, Pennsylvania

**Keywords:** Radiation, Proctitis, Ectasia, VEGF, CEACAM1

## Abstract

**Background and Aims:**

It has been recently proposed to change the nomenclature of “chronic radiation proctitis” (CRP) to “radiation-associated vascular ectasia” on the basis that signs of inflammation are rarely observed. We herein present data supporting the idea that inflammation is a critical step that initiates the process that culminates in the characteristic changes of CRP.

**Methods:**

In support of inflammation in the pathogenesis of CRP, we review the pertinent literature and publish our new results, including the role of amifostine treatment and proinflammatory factors (p38 MAP kinase, VEGF, and CEACAM1).

**Results:**

Immunohistochemistry from anterior rectal wall biopsies obtained in a prospective pilot study demonstrates that expression of VEGF and the downstream vascular effector CEACAM1 were elevated before radiotherapy and declined with time. We also show that MAP Kinase p38 expression usually precede the radiation. Fibrosis scores increase from baseline at 9 and 18 months, while vascular scores decrease at 18 months.

**Conclusion:**

The proposed new nomenclature should be held in obeyance until more supportive data are presented. Possibly, the best way to view CRP is as a continuum that may take one of three forms, inflammation-predominant, vasculopathy-predominant, or mixed.

## Introduction

It has been recently^1^ proposed to change the term “Chronic Radiation Proctitis” (CRP) to “Radiation Associated Vascular Ectasia” because of the notion that inflammation is not a representative component of CRP, which is mainly recognized by vascular ectasia. This is based on the observation that the most common pathologic manifestation appears to be vascular ectasia^2^ while inflammation is uncommon.

We believe that this viewpoint is erroneous and that basing nomenclature on a single end-stage finding ignores the continuum of pathogenesis that leads to the end stage. Similarly, most renal pathologists would argue that a biopsy of end-stage renal disease is not informative of the pathogenesis of the initial renal disease.^3^ We therefore refute this change of nomenclature and offer data and a conceptual framework to support our position. The pathophysiology reveals an inflammatory component,^2^ particularly in its early phases, and especially regarding epithelial injury; hence, “it” is an appropriate suffix. Over time, vascular abnormalities may predominate, with or without continuing inflammation. Inflammation may also wax and wane over time.^4^

Herein, we report results of studies we have conducted that provide more insight into the pathophysiology of CRP and support the role of inflammation.

## Materials and Methods

### Role of VEGF, CEACAM1, and MAP Kinase p38 in CRP

Twenty nine patients with early-stage prostate cancer, confined to the gland, were enrolled in a study to examine the effects of the radioprotector agent amifostine. This was a joint study conducted by the VHA affiliates of Harvard Medical School and Wayne State University and the positive-trending results were published^5^ centering on relief of symptoms (by questionnaire) and signs (by periodic sigmoidoscopy at 9 and 18 months vs baseline before radiation). Patients received total prostatic radiation doses of 70.2 Gy (20 patients) or 73.8 Gy (9 patients) using a 4-field axial technique. Intrarectal aqueous amifostine^6^ was instilled 30 minutes before treatment sessions for the first 15 days of radiotherapy with incremental doses, in serial patient cohorts, from 500 mg to 2500 mg. At the Detroit VA, an approved Institutional Review Board amendment allowed for performing biopsies. Flexible sigmoidoscopy (FS) was performed with anterior, posterior, left, and right rectal wall mucosal biopsies before therapy and at 9 and 18 months in most patients. Biopsies were graded visually by FS and by pathological findings. Sections of mucosa were labeled with an anti-VEGF monoclonal antibody (Pharmingen), allowing for comparison of later time periods with baseline values. Immunohistochemistry of 5-μm slices of paraffin-fixed forceps biopsies by the ABC technique allowed for evaluation of expression of VEGF, CEACAM1 (a downstream effector of VEGF kindly gifted by Christophe Wagner), and Adnab-9 1:50 (binding to p87 an InImS effector, at the time available from DakoCytomation Inc, Burlingame, California). Significant immunohistochemistry labeling cutoff was set at ≥ 1+. Arbitrarily set by pathology on an semi-quantitative intensity scale of 0–3 where 0 is no staining seen, 1+ is mild staining seen, 2+ is moderate staining seen 4+ strong staining seen. Extracts from fresh biopsies, as described previously,^7^ were reacted at 5-μg protein/well and reported as OD-background. Biopsies were also assessed for mucosal architecture, ulceration, fibrosis, and vascularity associated with radiotherapy.^8^ Development of CRP was correlated with demographics and dose of amifostine (range 500–2500 mg given as rectal enema before radiation treatments).^6^ Western blotting was performed as previously described with pp38 MAPK antibodies,^9^ a kind gift from Dr Rishi at the Detroit VAMC.

## Statistics

The parametric Student’s *t*-test was used to analyze the differences of means using an online statistical program (www.vassarstats.net). Nonparametric tests to analyze proportions used Chi-squared test, Fisher’s test (with approximation of Woolf if indicated), and Pearson test. Linear correlation coefficients (r) and significance testing were obtained by the analysis of the least-squares method. Two-sided *P* values were considered significant at the *P* < .05 level, but the effects of strong trends were considered at *P* < .1.

## Results

**Figure 1:**
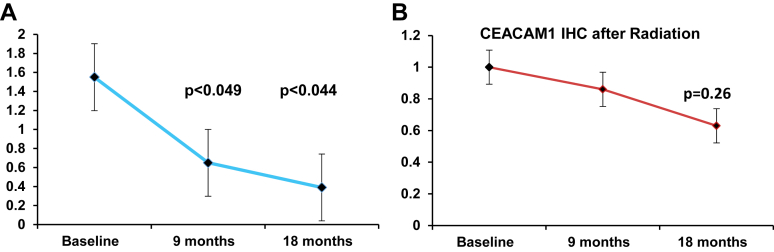
(A) Time line diagram with decreasing VEGF expression. (B) Time line diagram showing decreasing CEACAM1 expression.

At 18-m CRP (RTOG Grade 1) developed in 5/19 patients and we analyzed the associated findings.^10^ VEGF and to a lesser extent CEACAM1 were co-expressed at increased levels at baseline decreasing (VEGF *P* = .044; CEACAM1 *P* = .26) when scored over time (Figure 1A).

Error bars show ± standard error of the mean for the immunohistochemical score on ordinate and abscissa showing baseline before radiation with follow-up biopsies at 9 and 18 months. Levels are significantly reduced at 9 (*P* < .049) and 18 months (*P* < .044) (Figure 1B).

Error bars show ± standard error of the mean for the immunohistochemical score on ordinate and abscissa showing baseline before radiation and follow-up biopsies at 9 and 18 months. Unlike its upstream VEGF precursor expression, there are no significant reductions at 9 or 18 months (*P* < .26).

Younger age significantly predisposed to increased fibrosis; 59 ± 2 (> 1+) vs 72 ± 5.7 years (> 1+; *P* = .007) and mean scores were higher in younger patients < 65 years (1.67 ± 0.33 vs 1 ± 0 > 65 years; *P* < .034). Rectal telangiectasia at 18 months correlated with age > 65 years when compared to baseline (*P* = .024).

**Figure 2:**
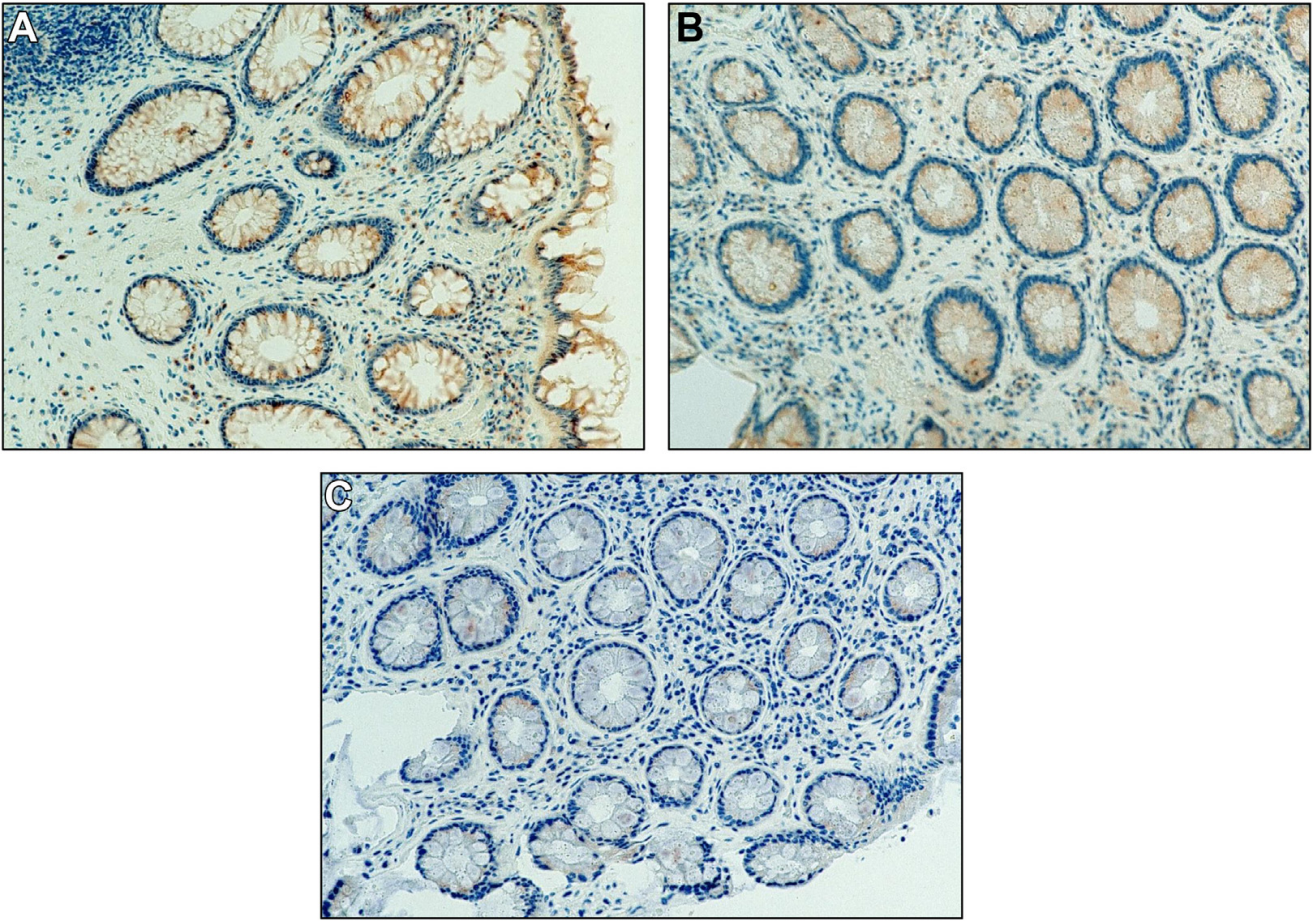
(A) VEGF immunohistochemistry at baseline. Photomicrographs show reddish-brown substrate uniformly in enterocytes with some focally intense staining in lamina propria. (B) VEGF immunohistochemistry at 9 months. Photomicrographs show very light reddish-brown uniform labeling in enterocytes and mild focal staining in lamina propria. (C) VEGF immunohistochemistry at 18 months. Photomicrographs uniformly demonstrate no obvious labeling in enterocytes or lamina propria.

High levels of VEGF were present at baseline (representative sections shown in Figure 2A), which declined somewhat in intensity at the same locations at 9 months (Figure 2B) and were still minimally detectable at 18 months with very low intensity with no stain observed in lamina propria (Figure 2A–C).

**Figure 3:**
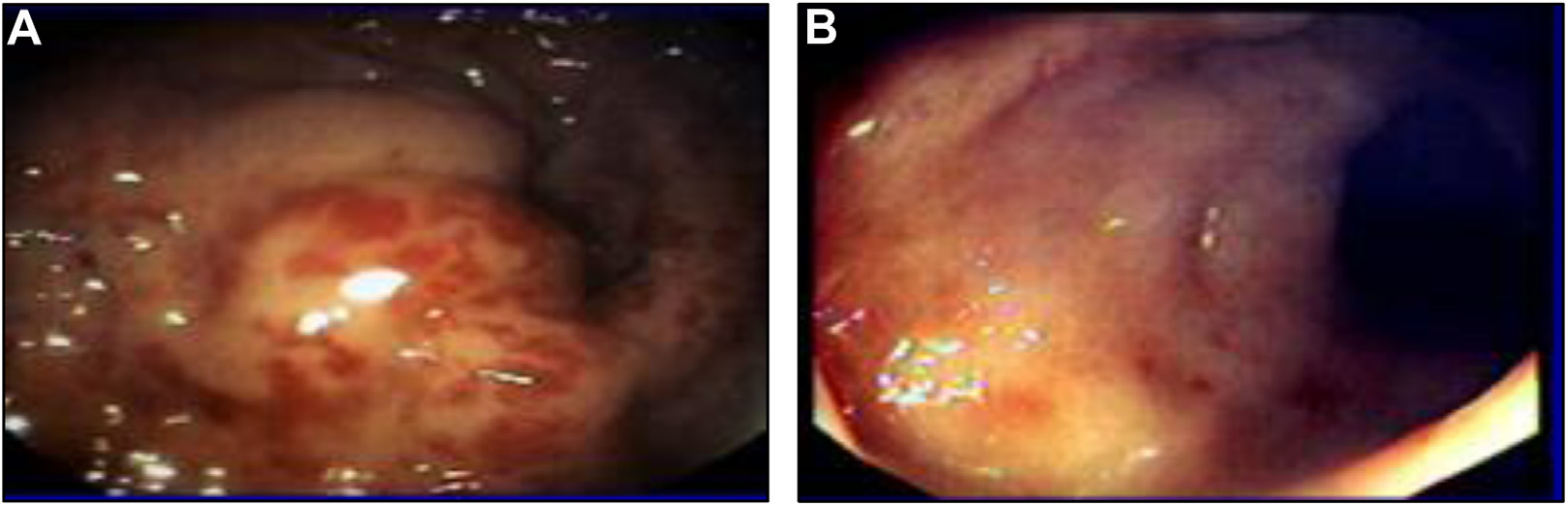
Endoscopic images of classic chronic radiation proctitis compared to a patient treated with amifostine. (A) shows an untreated rectal wall where vascular ectasia are prominent and (B) shows minimal changes after amifostine treatment.

A representative endoscopic image of classical vascular ectasia from a historical patient with Grade 1 RTOG/EORTC CRP is shown in Figure 3A, compared to a patient in Figure 3B who received amifostine showing minimal vascular ectasia (Figure 3).

**Figure 4:**
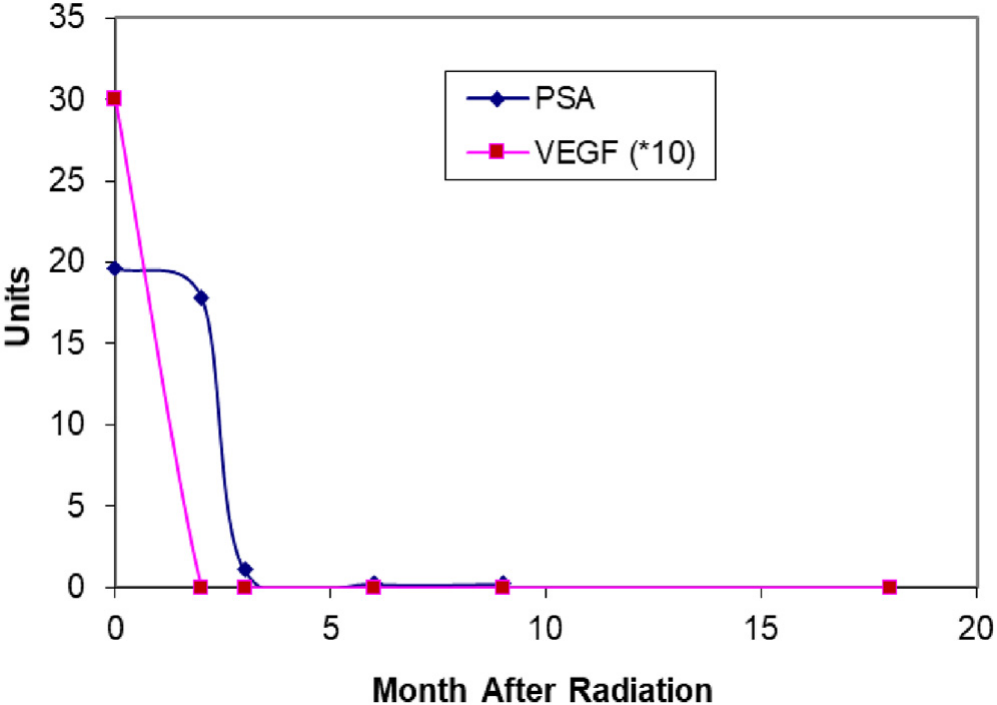
Dual line graph showing similar reduction of VEGF and PSA in a patient who received 1500 mg amifostine treatment. The VEGF IHC score levels are multiplied by 10 for better comparison. The ordinate depicts the level of IHC scoring and PSA levels at ng/mL and the abscissa, months after irradiation (0 = baseline).

Given that VEGF and its effector CEACAM1 are likewise expressed at baseline in the anterior rectal wall, we compared parallel PSA and VEGF decline over time as shown in Figure 4.

**Figure 5:**
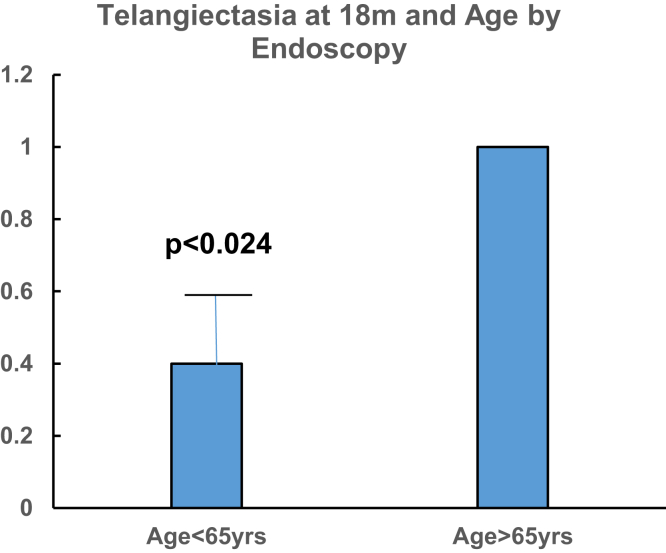
Bar diagram depicting a corresponding decrease of telangiectasia at 18 months in irradiated prostate patients with increased age.

At 18 months, younger patients (aged < 65 years) were more likely to have higher vascular scores (1 ± 0 vs 0.2 ± 0.44; *P* < .024, than older [> 65 years] as shown in Figure 5), but this did not necessarily increase the risk of CRP diagnosis (Figure 5).

Vascular ectasia observed at endoscopy was significantly less in the younger vs the older age group. This figure does not depict diagnosed chronic radiation proctitis.

**Figure 6:**
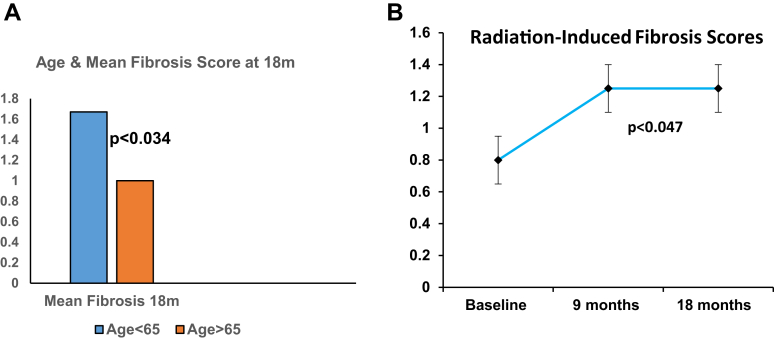
(A) Bar diagram showing the relation of age with microscopic fibrosis scoring. (B) Overall microscopic fibrosis score increases with time.

In comparison to vascular ectasia endoscopic scoring, fibrosis microscopic scores showed significantly increased fibrosis in the younger age group aged < 65 years as shown in Figure 6A.

Age was significantly correlated with the presence of the combined fibrosis score at 18 months in irradiated prostate cancer patients. Ordinate shows the fibrosis score, and abscissa the age group, which is color-coded (Figure 6B).

Figure 6B shows error bars ± standard error of the mean for the microscopic score on ordinate and abscissa show baseline scores before radiation with follow-up biopsies at 9 and 18 months. The fibrosis scores are significantly reduced at 9 and 18 months (*P* < .047).

Given the close proximity of prostate cancer to the anterior rectal wall,^11^ prostate cancer can cause matrix vascular mimicry and predispose the rectum to chronic radiation proctitis. We postulate that the practice of obtaining multiple prostate cores with access through the anterior rectal wall permits the creation of multiple sinus channels through which the prostatic vascular mimicry effectors^12^ can enter and suffuse the anterior rectal wall with resultant priming and enhancement of radiation effects causing the development of vascular ectasia. However, to invoke inflammatory effects, we need to introduce additional data below such as the effect of other anti-inflammatory or mitigating medications.

### Western Blotting of p38 MAPK at Baseline

**Figure 7:**
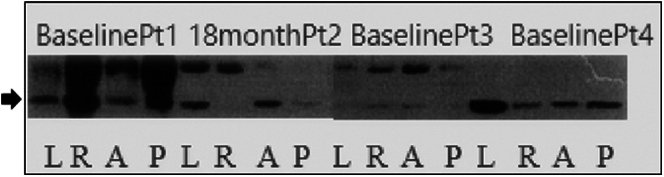
Western blot of 4 quadrant rectal biopsies reacted with an antibody for pp38 (arrow).

The MAP kinases are prime biomarkers of inflammation.^9^ We postulate that the inflammatory cascade occurring at the time of transrectal biopsy would be supported if MAPK would be expressed at that time, with radiation playing a later modulating role in the acute and chronic phases. We performed western blotting on 3 patients at baseline after prostate biopsy on sigmoidoscopic biopsy extract taken from the anterior, posterior, left, and right rectal walls. The pp38 (phosphorylated p38- indicated by the arrow in Figure 7) in 3 patients at baseline and patient 2 at 18 months show universal strong bands in 2 of the 3 patients at baseline and faint bands at anterior and right rectal walls in patient 3. The bands from patient 2 at 18 months postradiation shows a strong band in the anterior and left rectal walls (results shown in Figure 7). Two other patients, one at baseline and one at 1 year after radiation, had very faint bands (data not shown).

In animal studies inhibition of p38 MAP kinases reduced radiation senescence in mice, consistent with a role for these transmitters in aggravating radiation effects^13^ (Table).

**Table:** Summary Table

Parameter	Comparison	Baseline/age	Irradiation t	*P* value
Fibrosis score	BaseL vs 9 and 18 m	0.80 ± 0.42	1.25 ± 0.46	<.047
Vascular score	BaseL vs 9 m	0.53 ± 0.53	1 ± 0	<.013
Architectural score	BaseL vs 9 m ami+	1 ± 0	1.75 ± 0.50	<.025
VEGF-ami+	BaseL vs 9m	1.94 ± 1.57	0.42 ± 0.49	<.043
VEGF-ami+	BaseL vs 18 m	1.94 vs 1.57	0.30 ± 0.27	<.044
Vascular and age (yrs)	At 18 m	1 ± 0 (< 65)	0.2 ± 0.5 (> 65)	<.025
Telangiectasia and age	< 65 vs > 65 y	0.4 ± 0.18	1 ± 0	<.024

Student’s *t*-test analysis with comparison for means ± standard deviation.BaseL, baseline; m, months; vs, versus; yrs, years; ami+, high amifostine dose > 1000 mg; rr, relative risk.∗*P* = .06 (trend).

## Discussion

The presented data do not implicate VEGF or its downstream effector CEACAM-1^14^ in the process of CRP vasculopathy as the latter generally increases with time.

Given the intimate proximity of prostate cancer to the anterior rectal wall, prostate cancer effecting matrix vascular mimicry predisposes that organ to chronic radiation proctitis. We postulate that the practice of obtaining multiple prostate core biopsies with access through the anterior rectal wall permits the creation of multiple sinus channels through which the prostatic mimicry effectors enter and suffuse the anterior rectal wall with resultant priming and enhancement of radiation effects causing the development of vascular ectasia.

To understand the pathophysiology of CRP, we undertook a series of studies, the first with the radioprotector amifostine,^5^^,^^6^ where we found that the agent reduced symptoms of CRP. In other preliminary studies we found that aspirin and anti-inflammatory statins were significantly inversely related and protective against the development of CRP.^8^ In this study we studied factors associated with the development of CRP in prostate cancer patients. We specifically targeted potential contributions of medications taken concomitantly with radiotherapy because HMG-CoA reductase inhibitors (statins) and nonsteroidal anti-inflammatory medication (NSAIDs) have, respectively, been reported to enhance or reduce radiation effects in a number of organ systems.

From 1987–2002 there were 1177 prostate cancer patients diagnosed at our center and 63 patients were found to have endoscopy-proven CRP. Sixty one similarly diagnosed and irradiated prostate cancer patients with negative findings at endoscopy served as controls. Demographics, including body mass index, were compared. Of the study population, African Americans comprised 63% of the sample. Average age was 67.7 years, with median Gleason score of 7. There were 61 controls and 63 patients with CRP. Of the CRP patients, 19% were taking statins vs 55% of controls; 15% of CRP patients were taking NSAIDs vs 53% of controls. Logistic regression showed a decreased relative risk in patients taking statins (odds ratio [OR] 0.178, confidence interval [CI] 0.069–0.46, *P* = .0004) and in patients taking NSAIDs (OR 0.36, CI 0.15–0.82, *P* = .015). Multivariate regression analysis revealed that statins and NSAIDs were independent indicators for reduced risk of CRP (*P* = .0003 and *P* = .0158, respectively). Body mass index, aspirin, folic acid, and multivitamin medications were also analyzed but showed no significant differences were detected between the groups.

Statins and NSAIDs may protect the rectal wall by reducing radiotherapy-induced chemokines and COX-2 expression, respectively. These findings may prove clinically useful to reduce the incidence of CRP; prospective studies should be conducted to determine their clinical utility. In any event, it would appear that anti-inflammatory medications may be associated with a decrease in the incidence of CRP, lending credence to the importance of inflammation in CRP.

In the above mechanistic studies of vasculogenic factors, we found that VEGF and its downstream effector CEACAM1^8^ were already elevated at baseline before radiation and diminished with time suggesting that additional factors besides radiation contributed to the vascular ectasia.^12^ The above evidence does not implicate VEGF or its downstream effector CEACAM-1^13^ in the process of CRP vasculopathy as the latter generally increases with time.

Like melanoma and many cancers, prostate cancer causes vascular mimicry to divert blood-borne cellular-immunity effector cells, thereby escaping immune surveillance. Matrix vascular mimicry is also described where no vascular channels are formed but vasculogenic effectors such as VEGF are expressed as shown above. Given the intimate proximity of prostate cancer to the anterior rectal wall, we postulate that prostate cancer effects matrix vascular mimicry, predisposes the rectum to CRP, and this effect is preventable by amifostine, statins, and NSAIDs.

A most compelling argument in support of the continuum of the CRP process is that most long-term studies have demonstrated that acute radiation proctitis precedes the chronic phase and the initial proctitis severity often predicts the development of CRP.^11^ Acute radiation proctitis occurs in up to 75% of pelvic irradiated patients and its main characteristic is inflammation.^2^ Another argument for the importance of inflammation in CRP pathogenesis is the effectiveness of anti-inflammatory medication used to treat CRP regardless of whether the pathologist can detect signs of inflammation or not. These medications include corticosteroids via stabilization of neutrophil lysosomes and glucocorticoid receptor binding resulting in upregulation of genes opposing inflammation^13^; nonsteroid salicylate anti-inflammatory medications such as sulfasalazine or 5-ASA by impacting the prostaglandin system^15^ either alone or in combination; and short-chain fatty-acids^16^ which exert their effect through the inflammatory NFκB pathway. The final and most compelling reason is that while CRP is viewed as the hindmost part of the pathogenic continuum, the original response to events before, during, and after the delivery of the radiation dose can be shown to play an important pathogenic role.^17^ Indeed, some have classified CRP into three forms: (1) inflammation predominant (ICRP-I), (2) bleeding predominant (B-CRP), and (3) a mixed form with both features.^18^

In further support, we invoke elements of the innate immune system (InIMS)^19^ with radiation as the immune modulator, the phenomenon of vasculogenic mimicry,^12^ downstream effectors of the VEGF pathway, and the inflammatory p38 MAP kinases,^9^ all of which precede and engender vascular ectasia.

VEGF and its effector CEACAM1 are expressed at baseline in the anterior rectal wall and may depend upon amifostine dose. The trend of PSA and VEGF expression to decrease with time, shown above, may reflect a synergy between the presence of morphological features of aggressiveness of the prostate cancer and vasculogenesis at 18 m. Taken together, these data suggest that vascular mimicry may predispose to CRP and these effects may be mitigated by amifostine therapy. The prostate cancer may cause vascular mimicry to divert blood-borne cellular-immunity effector cells thereby enhancing survival.

We have addressed many components of inflammation and shown a role for each to a lesser or greater degree when CRP is viewed as a continuum with the starting point at the time of biopsy/diagnosis. With respect to vasculogenesis, we showed that VEGF and CEACAM1 are elevated before radiation and decline at 9–18 months after radiation by immunohistochemistry of anterior rectal wall biopsies and thus fibrosis secondary to inflammation is likely to account for the development of vascular ectasia. In their approach to CRP, workers from the Netherlands espouse 3 forms of chronic radiation injury, one of which is inflammatory predominant, the second most common but “self-limited”, vascular, and a third mixed,^18^ which supports the current nomenclature. The p38 western blots show that it is not only the anterior wall that express MAPK and these data endorse the view that any wall could be sampled as long it is performed consistently. They also point out the emerging interventions to manage the microbiome which we endorse when it is related to the primary role of Paneth cells.^19^ In their conclusions, they champion anti-inflammatory agents for treatment, among others. Japanese investigators have reproduced our work showing that VEGF in humans was not engendered by irradiation.^20^ They implicate other effectors such as metalloproteinase-8 (MMP-8), urokinase-type plasminogen activator (uPA), angiogenin, and fibroblast factor 1(FGF1) in 8 patients and 8 controls. They note that past animal work in rats did show an increase of VEGF at 3 months and TGFβ much earlier at 1 month, stimulating the fibrosis. The latter may be related to age as we have also documented fibrosis (as a result of inflammation), which tended to be more severe in younger patients as shown above. Irish authors point out that inhibition of endothelial apoptosis by FGF1 (chemical inhibitor) or by deleting the sphingomyelinase gene, abrogate radiation-induced injury, and death in mice.^13^ We have showed that anti-inflammatory medications can reduce the incidence of CRP and this has been supported in animal studies.^21^ There have been mixed results with NSAIDs to reduce CRP, but prostaglandin therapy has been reportedly successful.^22^ The role of MAPK, described above, is compelling from both the inflammatory role and the stress factor of irradiation that enhances endothelial apoptosis^23^ and likely sets the stage for CRP even before the tumor is irradiated.

There are mixed views on whether or not there is a role for chronic inflammation in radiation colitis. In most organ systems undergoing acute inflammation, chronic inflammation follows as part of healing. In colonic biopsies for acute radiation injury, mostly epithelial atypia, apoptosis, and sometimes eosinophils are seen.^24^ The injury may be more severe and result in ulceration or wound healing, wherever found in the body usually undergoes both acute and chronic phases to include both acute and chronic inflammation.^25^ It may be that pathologists hardly ever see subacute radiation colitis to include chronic inflammatory cells. Usually what is biopsied is a colon that is symptomatic, usually from bleeding, pain, or constipation, often from a more chronic injury. Thus, what is detected on biopsy is usually the chronic manifestations of radiation colitis, including hyalinization of the colonic lamina propria, regeneration of the mucosa, and telangiectasia of the capillaries. Accordingly, fibrosis has already occurred. Many mediators of fibrosis can be accounted for by endothelial cells and myofibroblasts, but there is also a role for chronic inflammatory cells causing fibrosis.^26^, ^27^, ^28^, ^29^, ^30^ Commensurate with the latter, we showed a continuum in the increase of the fibrosis score from baseline to 18 months without a parallel increase in the vascular score.

## Conclusion

We conclude that inflammation is an inseparable part of CRP,^31^ viewed as a continuum and that the current nomenclature should remain in place pending further research.
